# Cost-effectiveness analysis comparing ceftazidime/avibactam (CAZ-AVI) as empirical treatment comparing to ceftolozane/tazobactam and to meropenem for complicated intra-abdominal infection (cIAI)

**DOI:** 10.1186/s13756-019-0652-x

**Published:** 2019-12-21

**Authors:** Thitima Kongnakorn, Christian Eckmann, Matteo Bassetti, Eszter Tichy, Roberto Di Virgilio, Nathalie Baillon-Plot, Claudie Charbonneau

**Affiliations:** 1 Evidera, The Ark, 201 Talgarth Road, Hammersmith, London W6 8BJ UK; 2 Klinikum Peine, Academic Hospital of Medical University Hannover, Hannover, Germany; 3 Infectious Diseases Clinic, Department of Health Sciences, University of Genoa, Genoa and Hospital Policlinico San Martino IRCCS, Genoa, Italy; 4 Evidera, Bég u. 3-5 / 520, Budapest, 1022 Hungary; 5 Pfizer, Via Valbondione, 113, 00188 Rome, Italy; 6 Pfizer, 23-25 Avenue du Dr Lannelongue, 75014 Paris, France

**Keywords:** Economic model, Cost-effectiveness analysis, Ceftazidime-avibactam plus metronidazole, Ceftolozane/tazobactam plus metronidazole, Meropenem, Complicated intra-abdominal infection

## Abstract

**Background:**

The rising incidence of resistance to currently available antibiotics among pathogens, particularly Gram-negative pathogens, in complicated intra-abdominal infections (cIAIs) has become a challenge for clinicians. Ceftazidime/avibactam (CAZ-AVI) is a fixed-dose antibiotic approved in Europe and the United States for treating (in combination with metronidazole) cIAI in adult hospitalised patients who have limited or no alternative treatment options. The approval was based on the results of RECLAIM, a Phase III, parallel-group, comparative study (RECLAIM 1 [NCT01499290] and RECLAIM 2 [NCT01500239]). The objective of our study was to assess the cost-effectiveness of CAZ-AVI plus metronidazole compared with 1) ceftolozane/tazobactam plus metronidazole and 2) meropenem, as an empiric treatment for the management of cIAI in Italy.

**Methods:**

A sequential, patient-level simulation model, with a 5-year time horizon and 3% annual discount rate (applied to both costs and health benefits), was developed using Microsoft Excel® to demonstrate the clinical course of the disease. The impact of resistant pathogens was included as an additional factor.

**Results:**

In the base-case analysis, the CAZ-AVI sequence (CAZ-AVI plus metronidazole followed by a colistin + tigecycline + high-dose meropenem combination after treatment failure), when compared to sequences for ceftolozane/tazobactam (ceftolozane/tazobactam plus metronidazole followed by colistin + tigecycline + high-dose meropenem after treatment failure) and meropenem (meropenem followed by colistin + tigecycline + high-dose meropenem after treatment failure), had better clinical outcomes with higher cure rates (93.04% vs. 91.52%; 92.98% vs. 90.24%, respectively), shorter hospital stays (∆ = − 0.38 and ∆ = − 1.24 days per patient, respectively), and higher quality-adjusted life years (QALYs) gained per patient (4.021 vs. 3.982; 4.019 vs. 3.960, respectively). The incremental cost effectiveness ratio in the CAZ-AVI sequence was €4099 and €15,574 per QALY gained versus each comparator sequence, respectively, well below the willingness-to-pay threshold of €30,000 per QALY accepted in Italy.

**Conclusions:**

The model results demonstrated that CAZ-AVI plus metronidazole could be a cost-effective alternative when compared with other antibiotic treatment options, as it is expected to provide better clinical benefits in hospitalised patients with cIAI in Italy.

## Background

Complicated intra-abdominal infections (cIAI) arise due to perforation or necrosis of the gastrointestinal tract viscera [1]. Invasion of peritoneal and retroperitoneal space by bacteria further results in localized or diffuse peritonitis [2, 3]. According to a study by World Society of Emergency Surgery, the overall mortality rate was 10.5% [4]. Several Gram-negative bacteria, including *Escherichia coli, Klebsiella pneumoniae*, and the Enterobacter species, as well as other resistant pathogens, have been implicated in cIAIs and are believed to be accountable for more than 70% of the cases reported worldwide [5]. According to the United States (US) Centers for Disease Control and Prevention, some of the major Gram-negative bacteria that cause cIAIs and similar infections have developed resistance to currently available antibiotic drugs [6]. As bacterial resistance has increased, both the human and economic costs of treating resistant infections have risen, global concern has escalated, and the need to develop newer antibacterial agents has intensified [7].

Ceftazidime/avibactam (CAZ-AVI) is a novel, β-lactam/β-lactamase inhibitor fixed-dose combination drug containing ceftazidime (an established, extended-spectrum and avibactam (a unique, non-β-lactam, β-lactamase inhibitor) [1, 8]. CAZ-AVI has been approved by the European Commission and the US Food and Drug Administration (FDA) for the indication of cIAI (in combination with metronidazole) in adult patients [1, 9]. The introduction of CAZ-AVI is encouraging as this novel combination drug is known to have potent activity against several Gram-negative organisms, including some with multidrug resistance [9]. Hence, it could be a viable answer to rising incidence of resistance to most of the currently available antibiotic treatments that has not only become a challenge for clinicians to treat the disease but also has increased hospital stays as well as soaring healthcare costs related to cIAIs [3, 7].

Two identical, prospective studies (RECLAIM 1 [NCT01499290] and RECLAIM 2 [NCT01500239]) were conducted to evaluate the efficacy and safety of CAZ-AVI plus metronidazole compared to meropenem, followed by appropriate intravenous infusion, in hospitalised patients with cIAI. With the agreement of the US FDA and the European Medicines Agency (EMA), data from both these studies were combined to form a single inferential database [1].

Clinical cure at the test-of-cure (TOC) visit was the primary endpoint to assess the non-inferiority of CAZ-AVI plus metronidazole vs. meropenem. The assessment was performed in the microbiologically modified intention-to-treat population, and the modified intention-to-treat and clinically evaluable populations in the US and Europe, as requested by the FDA and EMA, respectively. RECLAIM was designed as a non-inferiority study. Meropenem, the current best practice drug for cIAIs, was selected as the comparator of CAZ-AVI.

In 2001, the Italian Group for Pharmacoeconomic Studies published guidelines for economic evaluations; these specify that cost-effectiveness assessments of newer agents in comparison to drugs already in the market should include the perspective of both healthcare providers and payers [10]. Economic evaluations (cost effectiveness and cost utility) are particularly important with new antibiotics such as CAZ-AVI, helping to establish their true market value when the interpretation of clinical trial results is limited by the increasing incidence of resistant pathogens [11, 12].

Italy is a top country in Europe with high prevalence of resistant Gram-negative pathogens. In 2014, *K. pneumoniae* isolates were resistant to carbapenems in approximately 25–50% of the cases; *P. aeruginosa* isolates were resistant to carbapenems in 25–50% of the cases and up to 10–50% of strains were classified as multi-drug resistant; *A. baumannii* had combine resistance to fluoroquinolones, aminoglycosides, and carbapenems in up to 50% of cases [13]. Furthermore, the antibiotics consumption out of hospitals was 27.8 doses per 1000 inhabitants, ranking the fifth country with high use of antibiotics in Europe [14].

The objective of this study was to analyse the cost effectiveness of CAZ-AVI plus metronidazole as an empiric treatment compared with meropenem or with ceftolozane/tazobactam plus metronidazole for appropriate hospitalised adult patients with cIAI from the Italian publicly funded healthcare (third-party payer) perspective.

## Methods

### Model structure

**Fig. 1:**
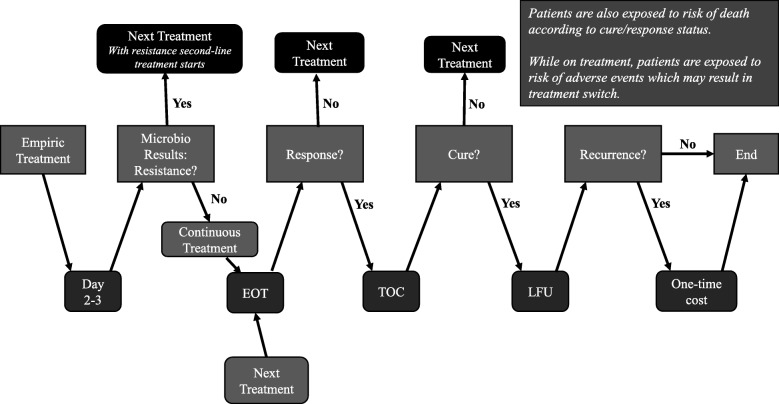
An overview of flow of each patient’s events. Abbreviations: *EOT* end-of-treatment visit, *LFU* late follow-up visit (42–49 days following the initiation of the treatment), *TOC* test-of-cure visit (28–35 days following the initiation of treatment)

A sequential, patient-level simulation model was developed in Microsoft Excel® to simulate the clinical course of cIAI, from diagnosis until clinical cure or death, after the initiation of empiric therapy. An overview of the model structure describing each patient’s pathway is shown in Fig. 1.

To begin with, 5000 patients hospitalised for cIAI were created in the model and every patient was assigned clinical characteristics using Monte Carlo sampling for type and resistance of infecting pathogen(s). These simulated patients were then duplicated in the model to create two identical sets of patients, to ensure that no factor other than the treatment had any influence on the outcome. The patients in one set received CAZ-AVI plus metronidazole as their empiric treatment, while those in the other received either meropenem, a widely used antibiotic for cIAI, or ceftolozane/tazobactam plus metronidazole, one of the newly approved antibiotics for cIAI at the time of this study.

Each patient entered the model on empiric treatment and the treatment was continued for the next 48–72 h (i.e., until microbiological results were available). If no resistance was observed in a patient’s test results, then the empiric treatment was continued (i.e., appropriate empiric treatment). Any patient whose test showed at least one pathogen resistant to the empiric treatment was then switched to the next treatment line and was counted as a clinical failure (Fig. 1).

At the end-of-treatment (EOT) visit, patients were assessed for response. If a response was achieved (i.e., appropriate empiric treatment), then the patient was subsequently assigned for assessment at the first follow-up visit (i.e., 28–35 days following initiation of treatment), equivalent to a TOC visit during a clinical trial. If no response was achieved (i.e., inappropriate empiric treatment), the patient was moved to the next treatment line and was counted as a clinical failure.

At the first follow-up visit, patients were assessed for clinical cure. If clinical cure was achieved, then the patient was subsequently assigned for assessment at the second follow-up visit (i.e., 42–49 days post-treatment initiation), equivalent to a long-term follow-up (LFU) visit in the clinical study. If clinical cure was not observed, the patient was switched to the next treatment line.

At the second follow-up visit, patients were assessed for recurrence of infection (equivalent to clinical failure observed at LFU visit in a randomized controlled study). If a patient had a recurrence, a one-time cost of recurrence was accrued; that cost was assumed to include all medical expenditures pertaining to management of the recurrence, such as cost of medication (e.g., antibiotics) and hospitalisation.

During the simulation of the clinical course, each patient was also exposed to risks of treatment-related adverse events (AEs) and in-hospital death. When an AE occurred, the medical cost of managing the AE was accrued and some patients may discontinue the AE-related treatment and switch to the next treatment or to best supportive care. All cost outcomes, life years (LYs) and quality-adjusted life years (QALYs) were accrued over the time horizon or until the patients died.

The model allowed inputs on resistance of pathogens to have impact on model outcomes. To incorporate the additional burden of antibiotic resistance (which cannot typically be captured in clinical trials), three assumptions were made in the base case. First, a 10% additional cost of hospitalisation was assumed to cover the increased utilization of resources to treat patients with antibiotic-resistant pathogens [14]. Next, the mortality rate for patients with antibiotic-resistant disease was assumed to be 20% higher compared to those patients who had no resistance but received inappropriate antibiotic therapy [14–16]. Last, based on clinical expert opinion, the efficacy of subsequent treatment was assumed to be decreased by 10%, as resistance to the empiric treatment may have impact on the subsequent treatments. All these three assumptions were tested in a scenario analysis.

### Treatment comparison

In the model, each sequence was composed of an empiric treatment followed by a second line of treatment, that was administered after the failure of the empiric treatment for any reason (e.g., resistance, lack of response or clinical cure, serious adverse event [SAE]). The treatment sequences were in accord with current treatment strategies in Italian clinical practice, based on clinical advice. Although some publications suggest that the monotherapy of colistin is not inferior to colistin combination therapy [17–19], these papers evaluate more broader spectrum of disease or other disease as our current evaluations and the data these publications use are not Italy-specific. Therefore, we relied on the opinion of clinical experts for the second-line treatment in our evaluation and a triple combination therapy of colistin + tigecycline + high-dose meropenem was selected. A scenario analysis was run using cost of colistin monotherapy instead of the triple combination in second-line, the results showed small increase in the incremental cost-effectiveness ratios (ICERs), they still remained below the threshold of €30,000 per QALY. There were a total of three treatment sequences: 1) empiric treatment with CAZ-AVI plus metronidazole, followed by a second-line treatment of a combination of colistin (intravenous) and tigecycline plus high-dose meropenem (colistin + tigecycline + high-dose meropenem), called the ‘CAZ-AVI sequence’; 2) empiric treatment with ceftolozane/tazobactam plus metronidazole, followed by colistin + tigecycline + high-dose meropenem (the ‘ceftolozane/tazobactam sequence’); and 3) meropenem alone, followed by colistin + tigecycline + high-dose meropenem (the ‘meropenem sequence’). Two treatment sequences were analysed at a time; the CAZ-AVI sequence vs. the ceftolozane/tazobactam sequence; and the CAZ-AVI sequence vs. the meropenem sequence.

### Model inputs and data sources

Model inputs were obtained primarily from clinical studies, published literature, and publicly available databases. Table 1 summarizes model inputs and data sources pertaining to baseline pathogens and their resistance rate for each treatment sequence. The analysis was based on the five most frequent baseline pathogens observed in the RECLAIM study. Inputs on resistance rates of CAZ-AVI plus metronidazole and meropenem were calculated in a forecast model obtained from 2017 resistance data for Italy [17, 20] and were validated by clinical experts.

**Table 1:** Baseline pathogens and resistance rate for each treatment sequence

Pathogens	Frequency of baseline pathogens^a^	Resistance rate by pathogens		
		CAZ-AVI + metronidazole^b^	Ceftolozane/Tazobactam + metronidazole^b^	Meropenem^b^
*Escherichia coli*	81%	1%	0%^c^	1%
*Streptococcus anginosus* group	15%	0%^c^	0%^c^	0%^c^
*Klebsiella pneumoniae*	13%	1%	52%^d^	52%
*Bacteroides fragilis*	12%	0%^c^	0%^c^	0%^c^
*Pseudomonas aeruginosa*	11%	7%	7%^e^	24%

CAZ-AVI = ceftazidime-avibactam.

^a^Five most frequently identified baseline pathogens in the RECLAIM study

^b^2017 resistance data for Italy, calculated from forecast model [17, 20], and expert opinion

^c^Assumption, due to lack of evidence

^d^Given that ceftolozane/tazobactam has no coverage for *K. pneumoniae* carbapenemase (KPC), the base case was assumed using the resistance rate of *K. pneumoniae* to carbapenems

^e^Expert opinion

Other model inputs and data sources are given in Table 2. Treatment efficacy was assessed by clinical evaluation at different stages, via response achieved at EOT visit, clinical cure achieved at TOC (i.e., first follow-up) visit, and recurrence of the infection observed at LFU (i.e., second follow-up) visit. Only SAEs which had relevant cost impact and may have resulted in treatment discontinuation or treatment switch were considered. Treatment efficacy and safety inputs for CAZ-AVI plus metronidazole and meropenem were obtained from the RECLAIM clinical trial, while those for ceftolozane/tazobactam plus metronidazole were sourced from published literature [35]; those same inputs for the second-line treatment of colistin + tigecycline + high-dose meropenem were based on expert opinion. Classification of in-hospital mortality of a patient was made depending on appropriateness of the empirical treatment and resistance to it. In-hospital death rates were sourced from published literature, as there were only small death counts observed in the clinical trials. Treatment duration inputs were based on product labels.

**Table 2:** Model inputs and data sources

	CAZ-AVI + metronidazole	Ceftolozane/Tazobactam + metronidazole	Meropenem	Colistin + tigecycline + high-dose Meropenem
Probability of clinical cure ^a^	91.7%^b^	94.1%^c^	92.5%^b^	75.0%^d^
Probability of AE^e^	4.9%^e^	8.1%^c^	3.8%^e^	14.8%^f^
Probability of recurrence	0.0%^b^	0.0%^g^	0.6%^b^	0.0%^g^
Treatment duration	9.5 days^h^	9.0 days^h^	9.5 days^h^	9.5 days^h^
Probability of in-hospital death^i^				
Appropriate empiric treatment: 4.80% Inappropriate empiric therapy: 10.70% Resistant to empiric therapy: 12.84%^j^				
Utility (quality of life)				
With clinical response: 0.92^k^ Without clinical response: 0.61^l^				
Hospital length of stay^m^				
With clinical response: 11.71 days Without clinical response: 24.13 days				
Proportion of hospitalisation days in ICU ^b^				
With clinical cure: 26.92% With clinical failure: 11.45%				
Daily drug costs,^n^ (average daily dose)	€ 300.77 (CAZ-AVI 7500 mg; metronidazole 1500 mg)	€ 248.97 (ceftolozane/tazobactam 1500 mg; metronidazole 1500 mg)	€ 55.32 (3000 mg)	€ 218.55 (colistin [IV] 5 mg; tigecycline 100 mg; meropenem 6000 mg)
Hospital cost per day		General ward: € 697.23^o^; ICU € 1383.00^p^		
Cost of SAE^o^		€ 3027		
Cost of recurrence^o^		€ 6787		

AE = adverse event; BNF=British National Formulary; CAZ-AVI = ceftazidime-avibactam; ICU = intensive care unit; IV = intravenous; SAE = serious adverse event.

^a^Probability of clinical cure of patients without resistance

^b^RECLAIM clinical study data [1]

^c^Solomkin et al. 2015 [35]

^d^Expert opinion

^e^AEs considered in the model included only serious AEs, as these have relevant cost impact and can result in treatment discontinuation or treatment switch. Probability of SAE (up to EOT) was based on RECLAIM clinical study data

^f^Pooled data from multiple sources: Chen et al. 2010 [21], Fomin et al. 2005 [22], Oliva et al. 2005 [23], Qvist et al. 2012 [24], and Towfigh et al. [25]

^g^Assumption (due to lack of data)

^h^European Medicines Agency (EMA) product labels [26, 27]

^i^Sturkenboom et al. 2005 [28]

^j^Assumed to be 20% higher than mortality among patients with inappropriate empiric therapy (without resistance)

^k^Song et al. 2012 [29]

^l^Delate et al. 2001 [30], assuming similar utility for patients with different infections. Deterministic sensitivity analysis showed small impact of utility of cIAI (i.e., utility applied while patients have not been cured) on the results

^m^Payer Analysis data

^n^AIFA, Agenzia Italiana del Farmaco. 2014 (except for cost of colistin which was taken from BNF, converted to Euros using an exchange rate of £1 = €1.36) [31]

^o^Italian hospital diagnosis-related groups (DRGs 2013 and 2015) [32, 33]

^p^Tan et al. 2012 [34]

The daily costs of CAZ-AVI, colistin + tigecycline + high-dose meropenem, and ceftolozane/tazobactam plus metronidazole were available on the official site of the Agenzia Italiana del Farmaco (AIFA), the Italian medicine agency [31]. However, the cost of colistin was not available in the AIFA database, so was taken from British National Formulary [36]. Hospitalisation costs in the model were calculated based on hospital length of stay and the proportion of time spent in intensive care unit vs. general ward. These inputs were categorized based on whether the patient achieved clinical cure. Costs of treatment-related SAEs were estimated as a one-time cost based on weighted average cost of various types of SAEs as observed in the RECLAIM study.

### Analyses

#### Base-case analysis

In the base-case analysis, a 5-year time horizon was considered to cover the episode of the infection and the long-term impact. A 3% annual discount rate was applied in the model to both costs and health benefits [10]. Two pairwise comparisons were performed to compare 1) the CAZ-AVI sequence vs. ceftolozane/tazobactam sequence and 2) the CAZ-AVI sequence vs. meropenem sequence.

#### Probabilistic sensitivity analysis

The robustness of outcome results with regards to model uncertainty was studied through probabilistic sensitivity analysis (PSA) by using second-order Monte Carlo simulation and running the model for 100 simulations. A probability distribution was assigned to each parameter (i.e., costs and outcomes) to generate the inputs and to calculate the cost and effectiveness outcomes of each treatment sequence. Costs relating to use of healthcare resources were assumed to follow gamma distributions, while inputs limited to between zero and one (like probabilities and utilities) were assumed to follow a beta distribution. The standard error for some of these parameters was assumed to equal 10% of the mean because of lack of information on their variability. Cost-effectiveness acceptability curves were obtained by plotting the data of the probabilistic analysis on the cost-effectiveness plane.

#### One-way deterministic sensitivity analyses

Key model parameters were identified using one-way deterministic sensitivity analyses (DSA), where each parameter was varied by ±20% of the base-case values while holding all other parameters constant. The results were defined in terms of incremental net benefit (INB), calculated as the difference of the incremental QALYs multiplied by the willingness-to-pay (WTP) threshold and the incremental costs, and were presented in the form of tornado diagrams.

#### Scenario analyses

Two scenario analyses were performed to test the assumptions used in the model. In one scenario (the ‘no resistance adjustments’ scenario), we removed the additional economic burden we had assumed in the base case to account for resistance to empiric antibiotics, such as an increase of in-hospital death rate, an increase in daily hospitalisation cost, and a decrease in efficacy of the second-line treatment. In the other (the ‘100% cure in second-line’ scenario), efficacy of the second-line treatment was set to 100% (i.e., assuming patients were switched to the ‘right’ treatment once the resistant pathogens were identified from microbiological results).

## Results

### Comparison: CAZ-AVI sequence vs. Ceftolozane/Tazobactam sequence

#### Base-case results

**Fig. 2:**
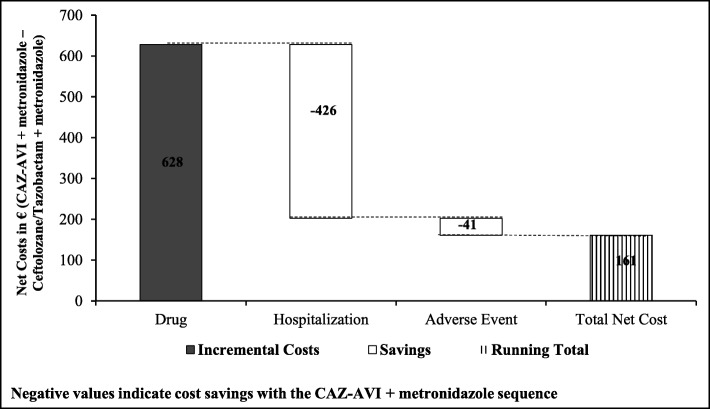
Incremental cost outcomes per patient for CAZ-AVI sequence vs. Ceftolozane/Tazobactam sequence. Abbreviations: *CAZ-AVI* ceftazidime-avibactam. CAZ-AVI sequence: CAZ-AVI plus metronidazole, followed by colistin + tigecycline + high-dose meropenem. Ceftolozane/tazobactam sequence: Ceftolozane/tazobactam plus metronidazole, followed by colistin + tigecycline + high-dose meropenem

Results of the key base-case analysis comparing the CAZ-AVI sequence with the ceftolozane/tazobactam sequence are presented in Table 3 and Fig. 2. The proportion of patients cured was comparable between CAZ-AVI sequence and ceftolozane/tazobactam sequence (93.04% vs. 91.52%, respectively). Consequently, a slightly lower proportion of in-hospital deaths due to infection was observed with the CAZ-AVI sequence, thus resulting in a slight increase in LYs and QALYs (0.027 LY and 0.039 QALY per patient) over the 5-year time horizon. A higher number of cures with the CAZ-AVI sequence led to a reduction in the average length of hospital stay (0.38 days per patient) (Table 3).

**Table 3:** Clinical and economic outcomes for CAZ-AVI sequence vs. Ceftolozane/Tazobactam sequence (discounted by 3%)

Outcomes	CAZ-AVI sequence^a^	Ceftolozane/Tazobactam sequence^b^
% of patients with cure	93.04%	91.52%
% of patients died in hospital	5.02%	5.60%
% of patients with AE	6.12%	7.48%
Average number of days in hospital	13.04	13.42
LYs	4.411	4.384
QALYs	4.021	3.982
Drug costs	€ 2952	€ 2324
Hospitalisation costs	€ 11,355	€ 11,781
SAE costs	€ 185	€ 226
Recurrence costs	€ 0	€ 0
Total costs	€ 14,492	€ 14,331
Incremental cost per QALY gained	€ 4099	

AE = adverse event; CAZ-AVI = ceftazidime-avibactam; LY = life year; QALY = quality-adjusted life year; SAE = serious adverse event.

^a^CAZ-AVI plus metronidazole, followed by colistin + tigecycline + high-dose meropenem

^b^Ceftolozane/tazobactam plus metronidazole, followed by colistin + tigecycline + high-dose meropenem

Results of cost outcomes suggested that there was an incremental cost of € 161 per patient with the CAZ-AVI sequence. Higher drug costs (estimated increase of € 628 per patient) were offset by a reduction in hospitalisation costs (estimated decrease of € 426 per patient). The costs of treatment-associated SAEs were also lower in the CAZ-AVI sequence (average decrease of €41) (Fig. 2). Overall, the ICER was estimated at € 4099 per QALY gained, which was well below the WTP threshold of € 30,000 per QALY in Italy (Table 3).

#### Scenario analyses results

In the ‘no resistance adjustments’ scenario, the ICER increased to € 11,461 per QALY. However, the CAZ-AVI sequence is still considered a cost-effective option given the WTP threshold of € 30,000 per QALY accepted in Italy. Results for the ‘100% cure in second-line’ scenario suggested that the assumption of 100% efficacy for the second-line treatment had no impact on the ICER, as the ICER changed by less than 1% compared to the base case (Table 4).

**Table 4:** Scenario analyses results for CAZ-AVI sequence vs. Ceftolozane/Tazobactam sequence

Scenario	Incremental cost per QALY gained (% change from base case)
Base case	€ 4099
Resistance adjustments	
No adjustments	€ 11,461 (+ 180%)
Second-line efficacy	
Assumed 100% response/cure rates in treatment	€ 4060 (− 1%)

CAZ-AVI = ceftazidime-avibactam; QALY = quality-adjusted life year.

CAZ-AVI sequence: CAZ-AVI plus metronidazole, followed by colistin + tigecycline + high-dose meropenem. Ceftolozane/tazobactam sequence: Ceftolozane/tazobactam, followed by colistin + tigecycline + high-dose meropenem

#### Deterministic sensitivity analysis results

**Fig. 3:**
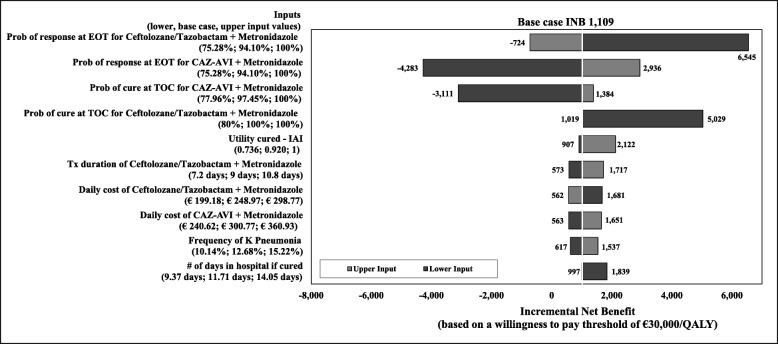
One-way deterministic sensitivity analysis for CAZ-AVI sequence vs. Ceftolozane/Tazobactam sequence, INB based on a willingness-to-pay threshold. Abbreviations: *CAZ-AVI* ceftazidime-avibactam, *cIAI* complicated intra-abdominal infection, *EOT* end-of-treatment, *INB* incremental net benefit, *Prob* probability, *TOC* test-of-cure, *Tx* treatment . CAZ-AVI sequence: CAZ-AVI plus metronidazole, followed by colistin + tigecycline + high-dose meropenem. Ceftolozane/tazobactam sequence: Ceftolozane/tazobactam plus metronidazole, followed by colistin + tigecycline + high-dose meropenem. Note: A positive INB indicates the CAZ-AVI sequence is cost effective compared to the ceftolozane/ tazobactam sequence, and vice versa

Results of the DSA comparing the CAZ-AVI sequence with the ceftolozane/tazobactam sequence are presented as a tornado diagram (Fig. 3). The 10 parameters that most influenced the INB (calculated based on a WTP threshold of € 30,000 per QALY) are included and presented in their order of the influence. In the base case, the CAZ-AVI sequence was estimated with an INB of € 1019, suggesting that the CAZ-AVI sequence was cost effective (i.e., positive INB indicates cost effectiveness) in comparison to the ceftolozane/tazobactam sequence. Results from the DSA showed that variation in response rates at the EOT assessment and the clinical cure rates at the TOC visit highly influenced the outcomes. The INB increased when response and cure rates for the CAZ-AVI sequence were increasing or decreasing those for the ceftolozane/tazobactam sequence. Other parameters had a moderate influence on the INB. The INB results became negative, (i.e., the CAZ-AVI sequence was not cost effective at the threshold of € 30,000 per QALY), when the cure and response rates of CAZ-AVI were decreased and when the response rate of ceftolozane/tazobactam increased by 10%.

#### Probabilistic sensitivity analysis results

**Fig. 4:**
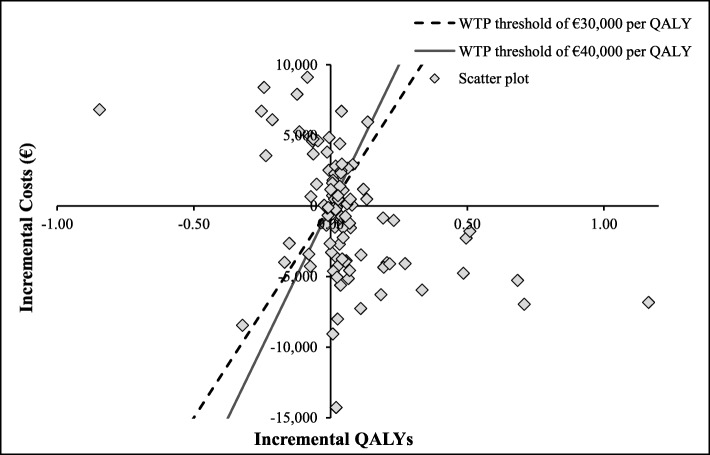
Results from probabilistic sensitivity analysis for CAZ-AVI sequence vs. Ceftolozane/Tazobactam sequence, on cost-effectiveness plane. Abbreviations: CAZ-AVI, ceftazidime-avibactam; QALY, quality-adjusted life year; WTP, willingness-to-pay . CAZ-AVI sequence: CAZ-AVI plus metronidazole, followed by colistin + tigecycline + high-dose meropenem. Ceftolozane/tazobactam sequence: Ceftolozane/tazobactam plus metronidazole, followed by colistin + tigecycline + high-dose meropenem. Notes: Each dot represents the cost-effectiveness outcome from each iteration. The threshold lines represent cost-effectiveness thresholds of € 30,000 or € 40,000 per QALY (i.e., the maximum amount society is willing to pay for a QALY gained). In cases that fall to the right and below this line, the CAZ-AVI sequence is cost effective compared to the ceftolozane/tazobactam sequence. In cases that fall to left and above this line, the CAZ-AVI sequence is not cost effective compared to the ceftolozane/tazobactam sequence

The PSA results showed that in 45% of the runs, the CAZ-AVI sequence was dominant, producing higher numbers of QALYs at lower costs compared to the ceftolozane/tazobactam sequence, as illustrated in Fig. 4 by the clustering of the majority of the iterations in the South-East quadrant. In 26% of the iterations, the CAZ-AVI sequence was found to be costlier but still produced a higher number of QALYs, with a second cluster in the North-East quadrant of Fig. 4.

**Fig. 5:**
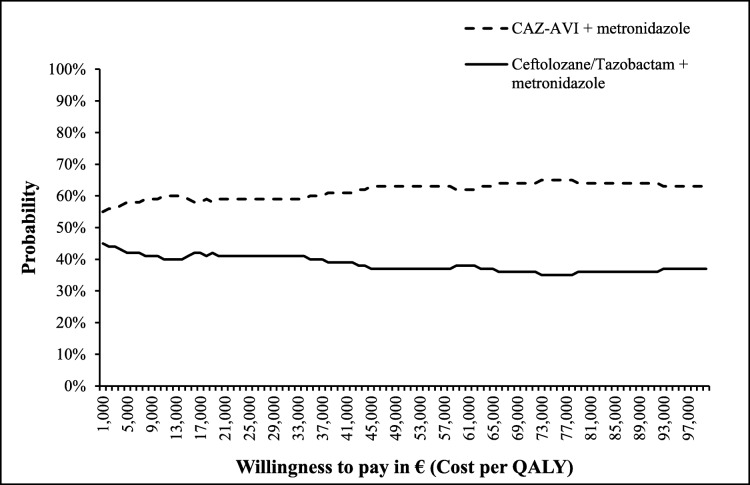
Results from probabilistic sensitivity analysis for CAZ-AVI sequence vs. Ceftolozane/Tazobactam sequence, on cost-effectiveness acceptability curve. Abbreviations: *CAZ-AVI* ceftazidime-avibactam, *QALY* quality-adjusted life year. CAZ-AVI sequence: CAZ-AVI plus metronidazole, followed by colistin + tigecycline + high-dose meropenem. Ceftolozane/tazobactam sequence: Ceftolozane/tazobactam plus metronidazole, followed by colistin + tigecycline + high-dose meropenem

The cost-effectiveness acceptability curves (CEAC) appearing in Fig. 5 suggests that the CAZ-AVI sequence had a higher percentage of being cost effective (ranging from 55 to 65%) compared to the ceftolozane/tazobactam sequence for all WTP thresholds explored (up to €100,000 per QALY).

### Comparison: CAZ-AVI sequence vs. Meropenem sequence

#### Base-case results

The model suggested better health outcomes for the CAZ-AVI sequence, with 92.98% of patients in the CAZ-AVI sequence achieving clinical cure compared to 90.24% of patients receiving the meropenem sequence. Time spent in hospital per patient was also lower in the CAZ-AVI sequence (∆ = − 1.24 days per patient) (Table 5). Patients treated with the CAZ-AVI sequence had higher number of QALYs as well (∆ = 0.059 QALYs per patient).

**Table 5:** Clinical and economic outcomes for CAZ-AVI sequence vs. Meropenem sequence, discounted by 3%

Outcomes	CAZ-AVI Sequence^a^	Meropenem Sequence^b^
% of patients with cure	92.98%	90.24%
% of patients died in hospital	5.04%	5.68%
% of patients with AE	6.14%	5.74%
Average days in hospital	12.94	14.18
LYs	4.410	4.381
QALYs	4.019	3.960
Drug costs	€ 2943	€ 818
Hospitalisation costs	€ 11,262	€ 12,453
SAE costs	€ 186	€ 174
Recurrence costs	€ 0	€ 22
Total costs	€ 14,391	€ 13,467
Incremental cost per QALY gained	€ 15,574	

AE = adverse event; CAZ-AVI = ceftazidime-avibactam; LY = life year; QALY = quality-adjusted life year; SAE = serious adverse event.

^a^CAZ-AVI plus metronidazole, followed by colistin + tigecycline + high-dose meropenem

^b^Meropenem, followed by colistin + tigecycline + high-dose meropenem

**Fig. 6:**
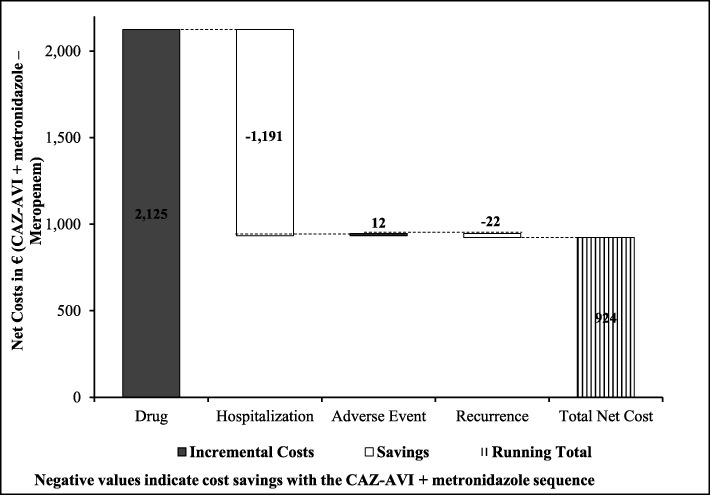
Incremental cost outcomes per patient for CAZ-AVI sequence vs. meropenem sequence. Abbreviations: *CAZ-AVI* ceftazidime-avibactam, CAZ-AVI sequence: CAZ-AVI plus metronidazole, followed by colistin + tigecycline + high-dose meropenem. Meropenem sequence: Meropenem, followed by colistin + tigecycline + high-dose meropenem

The average total drug costs with the CAZ-AVI sequence were higher by € 2125 per patient, mainly due to the higher drug acquisition cost of CAZ-AVI. However, the better clinical outcomes of the CAZ-AVI sequence meant that patients stayed in hospital an average of 1.24 days less than those receiving the meropenem sequence, reducing hospitalisation costs by € 1191 per patient. Overall, the net incremental cost was € 924 per patient in the CAZ-AVI sequence (Fig. 6). The ICER for the CAZ-AVI sequence was estimated at € 15,574 per QALY gained, again well below the € 30,000 per QALY WTP threshold accepted in Italy.

#### Scenario results

In the conservative scenario, when we removed the additional impact of resistance on hospitalisation costs, mortality, and cure rates of the subsequent treatment, the ICER was observed to increase to € 27,626 per QALY, still below Italy’s € 30,000 per QALY threshold. However, in the ‘100% cure in second-line’ scenario (in which the response/cure rates of second-line treatment were assumed to be 100%), the ICER increased to € 30,614 per QALY, slightly above the threshold (Table 6).

**Table 6:** Scenario analyses results for CAZ-AVI sequence vs. Meropenem sequence

Scenario	Incremental cost per QALY gained (% change from base case)
Base case	€ 15,574
Resistance adjustments	
No adjustments	€ 27,626 (+ 77%)
Second-line efficacy	
Assumed 100% response/cure rates in treatment	€ 30,614 (+ 97%)

CAZ-AVI = ceftazidime-avibactam; QALY = quality-adjusted life year.

CAZ-AVI sequence: CAZ-AVI plus metronidazole, followed by colistin + tigecycline + high-dose meropenem. Ceftolozane/tazobactam sequence: Ceftolozane/tazobactam plus metronidazole, followed by colistin + tigecycline + high-dose meropenem

#### Deterministic sensitivity analysis results

**Fig. 7:**
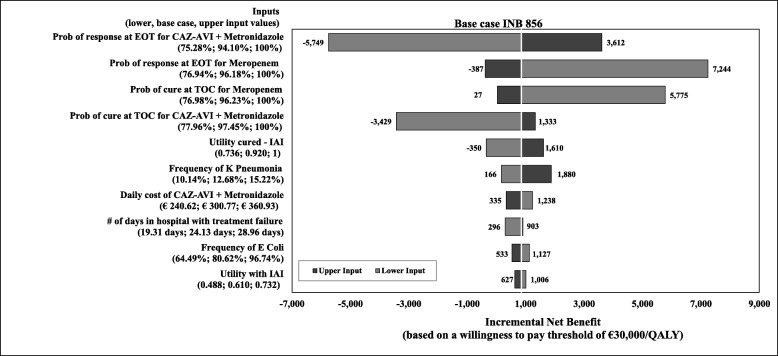
One-way deterministic sensitivity analysis for CAZ-AVI sequence vs. meropenem sequence, INB based on a willingness-to-pay threshold, Abbreviations: *CAZ-AVI* ceftazidime-avibactam, *cIAI* complicated intra-abdominal infection, *EOT* end-of-treatment, *INB* incremental net benefit, *Prob* probability, *TOC test-of-cure*. CAZ-AVI sequence: CAZ-AVI plus metronidazole, followed by colistin + tigecycline + high-dose meropenem. Meropenem sequence: Meropenem, followed by colistin + tigecycline + high-dose meropenem. Note: Positive INB indicates CAZ-AVI sequence was cost effective compared to meropenem sequence, and vice versa

Results of the DSA comparing the CAZ-AVI with the meropenem sequence are presented as a tornado diagram (Fig. 7), showing the 10 parameters that most influenced the INB (based on a WTP threshold of € 30,000 per QALY) presented in their order of influence. In the base case, the CAZ-AVI sequence was cost effective (with a positive INB of € 856) in comparison to the meropenem sequence. Results from the DSA showed that varying the response rates at the EOT assessment and the clinical cure rates at the TOC visit influenced the outcomes most. The INB was observed to increase when response and cure rates for CAZ-AVI sequence were increased or when those for the meropenem sequence were decreased. Also, among the 10 most influential inputs are the drug cost of CAZ-AVI plus metronidazole, the number of hospitalisation days with treatment failure, frequencies of *K. pneumoniae* and of *E. coli*, and utilities. The CAZ-AVI sequence was not cost effective (i.e., INB was negative) when we lowered utility score for ‘cure’ (from 0.92 in the base case to 0.74), the rate of response at EOT or of clinical cure at TOC for the CAZ-AVI sequence, and when we increased response rate at EOT for the meropenem sequence.

#### Probability sensitivity analysis results

**Fig. 8:**
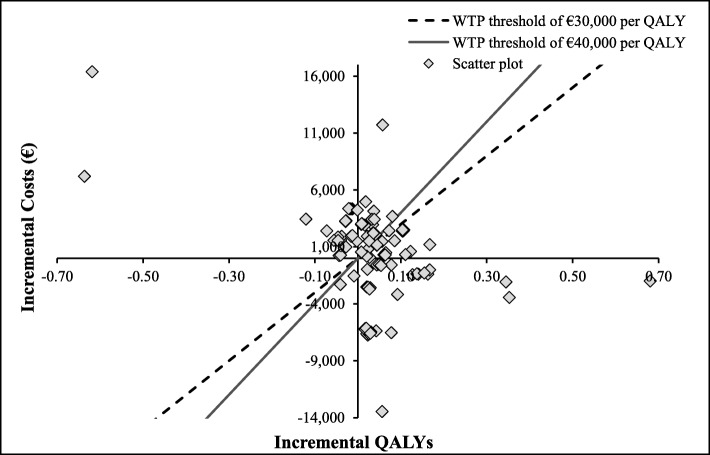
Probabilistic sensitivity analysis for CAZ-AVI sequence vs. meropenem sequence (on cost-effectiveness plane). Abbreviations: *CAZ-AVI* ceftazidime-avibactam, *QALY* quality-adjusted life year, *WTP* willingness-to-pay. CAZ-AVI sequence: CAZ-AVI plus metronidazole, followed by colistin + tigecycline + high-dose meropenem. Meropenem sequence: Meropenem, followed by colistin + tigecycline + high-dose meropenem. Notes: Each dot represents cost-effectiveness outcome from each iteration. The threshold lines represent cost-effectiveness thresholds (€ 30,000 or € 40,000 per QALY), the maximum amount society is willing to pay for a QALY gain. In cases that fall to the right and below this line, the CAZ-AVI sequence is cost effective compared to the meropenem sequence. In cases that fall to left and above this line, the CAZ-AVI sequence is not cost effective compared to the meropenem sequence

The PSA results showed that in 41% of the runs, the CAZ-AVI sequence was more effective, producing a higher number of QALYs with higher costs compared to the meropenem sequence, as illustrated in Fig. 8, where the majority of the iterations lie in the North-East quadrant. Additionally, the CAZ-AVI sequence was found to be dominant in 32% of the iterations (higher QALYs with lower costs; runs appear in the South-East quadrant) and found to be dominated in 25% of the iterations (lower QALYs with higher costs; runs appear in the North-West quadrant).

**Fig. 9:**
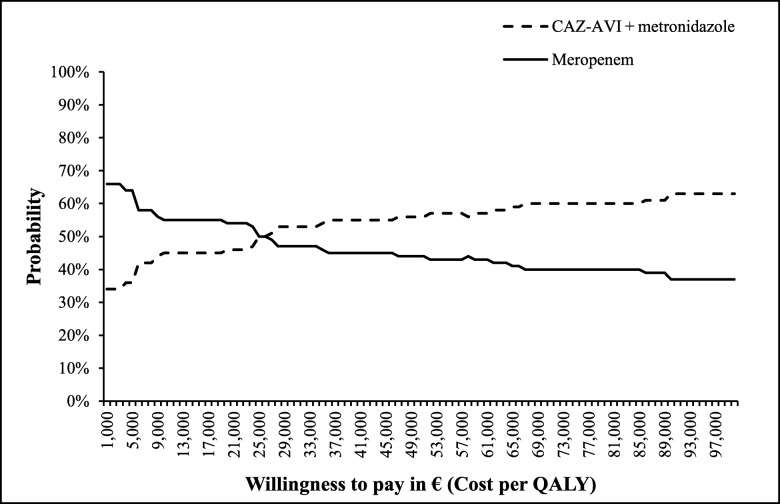
Probabilistic sensitivity analysis for CAZ-AVI sequence vs. meropenem sequence (on cost-effectiveness acceptability curve). Abbreviations: *CAZ-AVI* ceftazidime-avibactam, *QALY* quality-adjusted life year. CAZ-AVI sequence: CAZ-AVI plus metronidazole, followed by colistin + tigecycline + high-dose meropenem. Meropenem sequence: Meropenem, followed by colistin + tigecycline + high-dose meropenem

The CEAC in Fig. 9 suggests that the CAZ-AVI sequence had a higher probability of being a cost-effective treatment sequence compared to the meropenem sequence when the WTP threshold was at least € 26,000 per QALY.

## Discussion

The impact of bacterial resistance on the cost effectiveness of antimicrobial therapy in cIAIs has not been systematically investigated, perhaps due to a lack of evidence. In this study, we analysed cost effectiveness of CAZ-AVI plus metronidazole as an empiric treatment in comparison to ceftolozane/tazobactam plus metronidazole and to meropenem for hospitalised patients with cIAI from the Italian publicly funded healthcare (third-party payer) perspective.

The base-case analysis suggested that empiric therapy with the CAZ-AVI sequence for patients hospitalised with cIAI was cost effective compared to both comparator sequences in terms of efficacy and cost outcomes. Clinical efficacy outcome data demonstrated that patients treated with the CAZ-AVI sequence had better clinical outcomes (i.e., higher proportion of patients cured, increased QALYs per patient, and reduced average length of hospital stays) than did those patients treated with either comparator sequence. The observed higher incremental costs related to drug acquisition and SAEs with the CAZ-AVI sequence were partly offset by the lower hospitalisation cost compared to the comparator sequences. The total cost outcomes for the CAZ-AVI sequence were comparatively higher (€ 161 and € 924 incremental cost vs. the ceftolozane/tazobactam and the meropenem sequences, respectively). However, the ICERs for the CAZ-AVI sequence (€ 4099 vs. the ceftolozane/tazobactam sequence and € 15,574 vs. the meropenem sequence) were still well below the accepted WTP threshold in Italy of € 30,000 per QALY. Scenario analyses show, when changing inputs in favour of the comparators – i.e., resistance adjustment factors are not considered, or second-line efficacy is assumed to be 100%, the ICERs still remain below or exceed only by a bit the threshold of € 30,000. Probabilistic sensitivity results demonstrate similar conclusions. When varying the input parameters of the model, the majority of the outcomes shows cost-effectiveness of CAZ-AVI sequence. Furthermore, in 45% (compared to ceftolozane/tazobactam sequence) and in 41% (compared to meropenem sequence) of the outcomes the CAZ-AVI sequence is dominant. Thus, the results indicate that CAZ-AVI can be a cost-effective alternative in treating patients with cIAI. Furthermore, a recent budget impact study demonstrated that including CAZ-AVI in the hospital formulary for treatment of cIAI in Italy had only a minimal impact (0.74% increase over three years) on the total healthcare budget [37].

In Europe, the higher prevalence of multi-drug resistance in several pathogens, including the *Enterococcus* species, carbapenem-resistant *P. aeruginosa* and *Acinetobacter baumannii*, ESBL-producing *E. coli,* and *Klebsiella* species that are responsible for IAIs, has led to a corresponding increase in associated mortality and morbidity rates [38]. Hence, careful use of currently effective antimicrobial agents has become important to avoid and/or minimize emergence of resistance [38]. The results from our study indicate that using CAZ-AVI plus metronidazole as an empiric treatment among patients in the current resistance situation where resistance to CAZ-AVI has not yet developed resulted in patients receiving appropriate treatment earlier, and in better health outcomes overall. In addition, although not reflected in the economic outcomes of this study, the reduction in hospital stay provided by the CAZ-AVI sequence implicitly indicates additional bed days that hospitals can reallocate to other patients, and also a reduction in the risk of acquiring and/or transmitting new infections associated with prolonged hospital stay.

Our study is the first to look at the cost effectiveness of CAZ-AVI in cIAIs; however, several earlier models have examined cost effectiveness of antibiotics used in this indication. A patient-level simulation model published by Prabhu et al. in 2017 demonstrated that ceftolozane/tazobactam plus metronidazole was more cost effective and dominated piperacillin-tazobactam as an empiric treatment, due to lower total cost per patient ($ 44,226 vs. $ 44,811 respectively) and higher QALYs gained per patient (12.85 vs. 12.70), resulting in reduced length of hospital stay (∆ = 0.63 days) per patient [5]. In an earlier study, Barie et al. compared the economic benefits of cefepime plus metronidazole with those of imipenem-cilastatin in the treatment of cIAIs; that study showed the cost effectiveness of cefepime [39].

Our model analysis has some important limitations to note. First, we had to predefine the treatment pathways because of the model structure; hence, the choices for subsequent treatment for each individual patient could not be defined. Second, the de-escalation of treatment of patients who are susceptible to the empiric treatment was not considered in the study. If step-down therapy had been included in the model, the results would have favoured CAZ-AVI given higher proportion of patients was susceptible to CAZ-AVI and thus treatment costs would have been lower. Therefore, this assumption can be considered conservative. Third, since the RECLAIM study and other published literature served as our clinical data source for efficacy and SAEs, other methods of data synthesis, such as indirect treatment comparisons or mixed treatment comparisons could not be used. Fourth, the available clinical data (efficacy and safety) were based on multi-centre clinical studies and thus were assumed to be applicable to Italy. Fifth, as AEs were captured as an aggregate, the AE unit cost we calculated was dependent on the distribution of the AEs observed in the CAZ-AVI clinical studies. Hence, a similar distribution of AEs was assumed for the other treatment sequences in the current model analysis. As shown in the results of this study, AE costs contributed only 1 to 2% to the total costs. Thus, the impact of this assumption on the results was considered minimal. Sixth, the model assumes all deaths occurring in the model are during hospital stay. Death after hospitalisation was not considered, as it was assumed that patients were cured a priori to hospital discharge. In addition, given the short time horizon death due to background mortality was not considered. These assumptions were applied to all treatment groups and thus were not expected to have significant impact on the results. Seventh, due to no available data on the utility of patients with cIAI at the time of the study, the model utilized published utility data of other infectious disease [30]. This input was tested in sensitivity analysis and was fond to have small impact on the results (Fig. 3 and Fig. 7). And lastly, given the antibiotic resistance rate can evolve over time, the results from our study may not be generalizable in the future.

Our study has several strengths to note as well. One, the model included a broad range of inputs, such as response, cure, treatment duration, hospitalisation, AEs, and infection recurrence, resulting in more realistic outcomes. Two, the impact of resistant pathogens, which cannot be demonstrated in the randomized clinical trials, was incorporated in the model, allowing us to demonstrate the real value of the antibiotic agents. The model allowed pathogen resistance to affect daily hospitalisation costs, mortality, and cure rates of second-line treatment. In a conservative scenario analysis that did not take the impact of resistance into account, the CAZ-AVI sequence still demonstrated its cost effectiveness despite the increase in the ICERs (i.e., the ICERs increased to € 11,461 and € 27,626 per QALY in the comparison of the CAZ-AVI sequence to the ceftolozane/tazobactam and the meropenem sequences, respectively). Three, various efficacy aspects, including clinical response, cure, and recurrence were analysed in the model to delineate the required clinical and economical outcomes in detail. Four, the model uses a patient-level simulation, a well-established efficacy and economic model technique, whose ‘treatment switch’ allows the movement of patients from one treatment level to the next. This approach allows development of real-world models covering detailed treatment-related consequences (e.g., the impact of resistance). All these strengths allow the true economic value of CAZ-AVI to be captured in a way that the necessary non-inferiority design of antibiotic clinical trials cannot. The rationales for the non-inferiority design are not in question; the aim of clinical trials for antibiotics is not to identify superior treatments but to find those that are efficacious and safe for use as alternate treatments when existing agents are rendered ineffective by emerging antibiotic resistance.

Unlike drugs in other therapeutic areas such as oncology or cardiovascular disease, antibiotics do not seem to be economically valued at an appropriate level by society. CAZ-AVI and other novel antibiotics reduce mortality when used to treat life-threatening infections and therefore are lifesaving drugs [40].

## Conclusions

In deciding on treatment for cIAIs, healthcare providers must consider a variety of treatment-related parameters, including local resistance data, efficacy, risk of AEs, and the resource burden associated with managing the infection. Choosing the appropriate empiric treatment is of high importance, since early and effective treatment not only results in better clinical outcomes, but also extends the lifetime of antibiotic agents in this period of rising pathogen resistance. The results from our study support that the combination of CAZ-AVI plus metronidazole is a suitable alternative to ceftolozane/tazobactam plus metronidazole and to meropenem, as it is expected to provide comparatively better clinical benefits (i.e., higher cure rates, shorter hospital stays, and improved quality of life) in a cost-effective manner for Italian patients with cIAI.

## Data Availability

The datasets used and/or analysed during the current study are available from the corresponding author on reasonable request.

## Abbreviations

CAZ-AVI: Ceftazidime/avibactam
cIAIs: Complicated intra-abdominal infections
EMA: European Medicines Agency
TOC: Test-of-cure
US: United States
EOT: End-of-treatment.
LFU: Long-term follow-up
AE: Adverse events
LYs: Life years
QALYs: Quality-adjusted life years
SAE: Serious adverse events
AIFA: Agenzia Italiana del Farmaco
PSA: Probabilistic sensitivity analysis
DSA: Deterministic sensitivity analyses
INB: Incremental net benefit
WTP: Willingness-to-pay
ICER: Incremental cost-effectiveness ratio
CEAC: Cost-effectiveness acceptability curves
